# Structural severity versus clinical reality: Are we measuring the right endpoints?

**DOI:** 10.1002/jeo2.70741

**Published:** 2026-05-10

**Authors:** Lika Dzidzishvili, Jorge Chahla

**Affiliations:** ^1^ Department of Orthopaedic Surgery and Traumatology, Knee Surgery and Arthroscopy Unit. Hospital Universitari Germans Trias i Pujol, Universidad Autónoma de Barcelona, Institut d'Investigació en Ciències de la Salut Germans Trias i Pujol (IGTP) Carretera de Canyet s/n, Badalona Barcelona Spain; ^2^ Department of Orthopaedic Surgery, Sports Medicine Division, Rush University Medical Center Midwest Orthopaedics at Rush, 1620 W Harrison St Chicago Illinois USA

**Keywords:** clinical outcomes, knee surgery, patient‐reported outcome, sports medicine, treatment success

## Abstract

Patient‐reported outcome measures are well established in sports medicine as a standard tool for assessing treatment success. Despite efforts to define clinically meaningful thresholds, studies often report improved clinical outcomes alongside suboptimal structural, biological or imaging findings. This evidence highlights a persistent gap between patient‐reported improvement and objective changes, underscoring the limitations of current outcome metrics. These findings challenge the assumption that better clinical outcomes translate into improved structural results. Reliance on subjective measures alone seems insufficient to define true success. A more comprehensive approach is needed to better align clinical assessment with biological and biomechanical realities and to more accurately define meaningful patient benefit.

**Level of Evidence:** N/A.

**Abbreviations**
ACLR: anterior cruciate ligament reconstruction
DB: double‐bundle
OA: osteoarthritis
PROMs: patient‐reported outcome measures
SB: single‐bundle

Patient‐reported outcome measures (PROMs) have been extensively used in sports medicine for decades and are widely accepted and well‐documented in the literature as a standard tool for evaluating treatment success [19, 21]. While many researchers have dedicated efforts to better define clinically important outcome values, such as the minimal clinically important difference and the patient‐acceptable symptom state, studies often reveal improved clinical outcomes despite suboptimal structural, biological, or imaging outcomes [8, 13, 22].

To keep this overview concise, we first examine the clinical, radiological, and biological outcomes associated with commonly performed knee procedures in sports medicine.

The field of anterior cruciate ligament reconstruction (ACLR) has evolved considerably over time, with a substantial body of literature addressing surgical techniques and graft selection for both primary and revision procedures. While long‐term radiographic data consistently demonstrate greater osteoarthritis (OA) progression in reconstructed knees [9, 11], PROMs have remained favourable across studies [10, 18], highlighting a persistent gap between structural changes and clinical perception.

The debate over single‐bundle (SB) versus double‐bundle (DB) ACLR for restoring knee stability and function remained a point of contention for decades. Numerous clinical and biomechanical studies have compared these techniques, with a key synthesis of findings presented in a systematic review of overlapping meta‐analyses [17]. The authors concluded that DB ACLR provided significantly better knee stability, assessed by KT arthrometry and pivot‐shift testing, compared to SB ACLR [17] However, this biomechanical advantage did not translate into superior clinical outcomes or a reduced risk of graft failure [17]. While the clinical relevance of improved biomechanics remains uncertain, an important question arises: are these biomechanical differences meaningful for patients? This discussion is far from settled. However, as surgeons, we must ask ourselves what truly matters for both our patients and for clinical decision‐making. Is it improved biomechanics, or are clinical outcomes the ultimate priority? The answer is both, but above all, graft failure remains the most critical concern for both patients and surgeons.

Beyond graft failure, the question of long‐term joint preservation remains equally compelling. Despite clear biomechanical differences between techniques, current evidence suggests that these do not consistently translate into meaningful clinical advantages, particularly with respect to OA progression [3].

A similar pattern emerges when examining graft maturation and adjunct procedures. While advanced imaging and biomechanical assessments demonstrate improved graft remodelling, enhanced rotational stability, and even reduced failure rates with combined augmentation procedures, these benefits are not consistently reflected in PROMs [16, 20, 24]. Indeed, the relationship between graft biology, structural integrity, and clinical performance remains complex and, at times, paradoxical, with imaging findings often failing to correlate with functional results [16].

This discordance is also evident in graft selection. While autografts demonstrate superior durability, with lower failure rates compared to allografts, these structural advantages do not translate into meaningful differences in PROMs [6, 12]. Together, these findings underscore a persistent discordance between biomechanical success and clinical benefit, challenging the assumption that superior postoperative clinical outcomes will translate into improved structural integrity.

Remnant preservation during ACLR has been advocated to enhance graft healing and improve knee stability [1, 7]. While available evidence demonstrates biological advantages of this technique, objective improvements are not reliably mirrored by clinically meaningful differences in PROMs, with most functional scores remaining comparable and observed differences often falling below established thresholds of clinical relevance or statistical significance [7].

Another relevant consideration is the role of partial meniscectomy in degenerative meniscal tears with pre‐existing OA. Although short‐ to mid‐term PROMs may show meaningful improvement in appropriately selected patients, these clinical gains are not consistently accompanied by structural preservation [2, 23]. Comparative evidence suggests that, while outcomes and failure rates may be similar between partial meniscectomy and repair in certain tear patterns, meniscectomy is associated with greater progression of compartmental degeneration [2]. Together, these findings reinforce the growing observation that clinical improvement does not necessarily translate into structural joint benefit.

Despite advances in meniscal root repair techniques, degenerative changes continue to progress, often in apparent contrast with persistently improved PROMs [5, 14]. Available evidence shows that although medial meniscus posterior root repair is associated with lower rates of OA progression and conversion to arthroplasty compared to meniscectomy, radiographic deterioration still occurs in a substantial proportion of patients, while clinical scores remain largely comparable between groups [4, 5, 14, 15]. This persistent divergence between imaging and symptoms, further supported by studies reporting ongoing cartilage degeneration despite clinical improvement, underscores the complex and not fully understood relationship between structural joint changes and perceived functional benefit.

As a final reflection, while the ultimate goal of every surgical intervention remains the improvement of patients' function and quality of life, reliance on subjective measures alone is insufficient to capture true success. The growing body of evidence highlights a persistent gap between patient‐reported improvement and objective structural and biomechanical changes, underscoring the limitations of current outcome metrics. Moving forward, a more comprehensive and integrative approach is needed, one that better aligns clinical assessment with biological and biomechanical realities and more accurately defines what constitutes meaningful benefit for our patients.

## CONFLICT OF INTEREST STATEMENT

Lika Dzidzishvili reports a relationship with European Society of Sports Traumatology, Knee Surgery and Arthroscopy (ESSKA); International Society of Arthroscopy, Knee Surgery, and Orthopaedic Sports Medicine (ISAKOS): Board or committee; Journal of Experimental Orthopaedics (JEO): Associate Editor Jorge Chahla reports a relationship with American Orthopaedic Society for Sports Medicine (AOSSM): Arthroscopy Association of North America (AANA): International Society of Arthroscopy, Knee Surgery, and Orthopaedic Sports Medicine (ISAKOS): Board or committee member. Paid consultant, presenter or speaker of Arthrex, CONMED Linvatec, and Smith & Nephew.

## ETHICS STATEMENT

The authors have nothing to report.
